# Silencing LINC00491 Inhibits Pancreatic Cancer Progression through MiR-188-5p-induced Inhibition of ZFP91

**DOI:** 10.7150/jca.65071

**Published:** 2022-03-21

**Authors:** Zhi Fang, Bin Ruan, Min Zhong, Jianping Xiong, Yanxia Jiang, Zhiwang Song

**Affiliations:** 1 Department of Oncology, the First Affiliated Hospital of Nanchang University, Nanchang, Jiangxi province, People's Republic of China.; 2 Jiangxi Key Laboratory for Individualized Cancer Therapy, Nanchang, Jiangxi province, People's Republic of China.; 3 Department of Rehabilitation Medicine, the First Affiliated Hospital of Nanchang University, Jiangxi province, People's Republic of China.

**Keywords:** pancreatic cancer, LINC00491, miR-188-5p, ZFP91, carcinogenesis

## Abstract

**Background:** Pancreatic cancer is recognized as one of the most malignant tumors with poor prognosis. Recently, long noncoding RNAs (lncRNAs) are considered as a potential prognostic biomarker of PC. However, the concrete biological effect of lncRNAs in PC remains unmasked. Herein, we explored the mechanism of LINC00491 in PC.

**Methods:** Quantitative real-time PCR (qRT-PCR) was administrated to detected the expression of LINC00491 in PC tissues and cell lines. Loss-of-function experiment *in vitro* and *in vivo* was carried out to figure out the biological effects of LINC00491 on PC carcinogenesis. Luciferase reporter assay, subcellular fractionation, western blotting, pull-down assay and RNA immunoprecipitation assay were further used to explore the mechanism of PC tumorigenesis of LINC00491.

**Results:** An increase of LINC00491 was detected in PC cell lines and tissues. Silencing LINC00491 *in vitro* and *in vivo* subsequently hindered cell migration, invasion, proliferation and tumor growth, respectively. Further research confirmed a negative and a positive connection of LINC00491 with microRNA 188-5p (miR-188-5p) level and Zinc finger protein 91 (ZFP91), a target of miR-188-5p, respectively. qRT-PCR and western blotting found that miR-188-5p was upregulated while LINC00491 was downregulated, concomitant with ZFP91 decreasing in PC cells or node mouse tumors, which could be significantly restored by inhibiting miR-188-5p. Besides, overexpression of miR-188-5p partially restored the inhibitory effect of LINC00491 diminution on proliferation, migration, and invasion of PC cells.

**Conclusion:** LINC00491 promotes PC proliferation, migration, invasion via the miR-188-5p/ZFP91 axis, suggesting LINC00491 could be a new therapeutic target for PC.

## Introduction

Pancreatic cancer (PC) is the fourth leading fatal malignant solid tumor in the United States, which could contribute to thousands of cancer-related deaths around the world 1. With the characteristics of high malignancy, morbidity and mortality, PC has become the research focus of scientists all over the world in recent years 2. Despite the significant advances in diagnostic approaches and systemic therapies for advanced disease over the past decades, the outcomes of PC patients were still unsatisfactory 3. Surgery and adjuvant chemotherapy are presently acknowledged as the best treatment strategy for patients with early PC, however, approximately 80% of patients diagnosed firstly at an advanced stage unfortunately lose the chance for curative resection 4. Hence, it is imperative to uncover underlying molecular mechanism and seek a new therapeutic target for treating PC.

Long non‑coding RNAs (lncRNAs) which are initially recognized as the “noise” of genomic transcription are a group of evolutionarily conserved RNA transcripts with over 200 nucleotides in length 5. Intriguingly, accumulating studies have elucidated that lncRNAs involved in almost every step of gene life cycle 6 and subsequently functioned in many biological and pathological courses, such as development 7, differentiation 8 and even oncogenesis 9, 10, etc. In addition, many lncRNAs have been revealed to be closely involved in occurrence and progression of PC, which exerts oncogenic or anti-oncogenic role to regulate the malignant characteristic of PC 11-13. MicroRNAs (miRNAs/miRs), a family of noncoding RNA transcripts with 17-24 nucleotides, participate in either downstream target mRNAs degradation or translational suppression 14, 15. The aberrant expression of miRNAs was also found to be relevant to the development of many diseases, such as diabetes 16, neurodegenerative diseases 17 and human cancers 18, etc. Although mounting researchers confirmed the competing endogenous RNA (ceRNA) hypothesis which suggested lncRNA can act as a “sponge” to competitively bind to miRNAs, thus sparing the negative regulation of miRNAs on their target 19, the relationship between lncRNA and miRNA remained to be unveiled.

Long intergenic non-coding RNA 491 (LINC00491) is a newly identified lncRNA located on human chromosome 5q21.1 20. Previously, evidences have shown that the aberrant expression of LINC00491 are relevant to the oncogenesis and progression of breast cancer 21, endometrial cancer 22, colon adenocarcinoma 23, 24 and esophageal squamous cell carcinoma 25. Furthermore, Zhang et al. 26 also illustrated LINC00491 can competitively bind to microRNA 324-5p (miR-324-5p) to promote the progression of non-small-cell lung cancer (NSCLC). However, the function of LINC00491 in other tumors including PC remains exactly vague.

In our study, we determined the expression and carcinogenic functions of LINC00491 in PC. LINC00491 aggravated pancreatic cancer progression by microRNA 188-5p (miR-188-5p)/Zinc finger protein 91 (ZFP91) axis, which may open up new avenues for the development of prevention and treatment of PC in the future.

## Materials and methods

### Clinical samples

24 paired PC tissues and adjacent normal tissues used in our study were obtained with written informed consent acquired under the requirements of the Ethics Committee of the First Affiliated Hospital of Nanchang University. The inclusion and exclusion criterion are as follows: The patients recruited in our study were ranged from 18 to 75 years old who were diagnosed as primary pancreatic cancer without any radiotherapy and chemotherapy and with no distant and/or multiple metastasis.

### Cell lines and cell culture

The cells used in our research such as normal human pancreatic ductal epithelial cells (HPDE), AsPC-1, BxPC-3, Capan-2, MIAPaCa-2, PANC-1 and SW1990 were purchased from the Chinese Academy of Sciences Cell Bank (Shanghai, China). The complete Dulbecco's Modified Eagle Medium (DMEM) contain 10% Fetal Bovine Serum (FBS) attained from Biological Industry (Kibbutz Beit Haemek, Israel) was utilized to cells culture.

### Quantitative real-time PCR (qRT-PCR)

The total RNAs of PC tissues and cells were extracted with Trizol reagent (TaKaRa, China). Subsequently, the PrimeScript RT Reagent Kit and the SYBR Premix Ex Taq purchased from TaKaRa company were further utilized to detect the specific molecular levels. GAPDH was used as an internal control. The experiment was repeated in triplicate and comparing the quantitative cycle (2^-ΔΔCq^) method was used to calculate the results 27. The primer lists are shown in Table 1 below.

### Cell transfection

The matched negative controls and siRNAs of LINC00491 were synthesized from GenePharm (Shanghai, China). Additionally, the scrambled short-hairpin RNA (sh-RNA) and LINC00491 knockdown plasmid which could steadily suppress LINC00491 were also obtained from GenePharm company. The miR-188-5p mimics and inhibitor, and the matched control (inhibitor-Con and miR-Con) were attained from GenePharm (Shanghai, China). A 50-70% density of cells was required while transfecting the above reagents into cells via Lipofectamine 3000 (Invitrogen). Next, the cells were used for further experiments after 48h-culturement.

### Cell proliferation assay

The proliferation abilities of PC cells were evaluated by the Cell Counting Kit-8 purchased from Glpbio company. Average 2×10^3^ cells were seeded into each well of 96-well plates overnight. Then 10 µl CCK-8 solution was added and cultured for 30 minutes, then the viabilities were measured for four consecutive days at 450 nm absorbance.

### Colony formation assay

500 cells planted in 6-well plates were washed with PBS after a 12-day incubation. Subsequently 4% paraformaldehyde and crystal violet were applied to fix and stain the treated cells for 20 minutes, respectively. Then the colonies were counted and finally compared after being washed with running water.

### Transwell assays

The migration and invasion assay were carried out as described previously 28. While the upper compartment contained 3×10^4^ cells mixed in 200 µl serum-free medium, the lower chamber was full of 600 µl complete medium after filling with 60 µl Matrigel (Corning, USA) for invasion assays and without for migration assays, respectively. After 48h-incubation, fixed with 4% paraformaldehyde and stained by crystal violet for separate 20 minutes, the cells were eventually photographed and recorded.

### Subcellular fractionation assay

The PARIS^TM^ Kit (AM1921) purchased from Thermo Scientific company was employed to isolate nuclear and cytoplasmic RNAs as described previously 29. Briefly, 2×10^6^ fresh cultured cells were washed once in PBS and placed on ice. Then the Cell Fractionation Buffer and Cell Disruption Buffer were used to split the sample for RNA isolation. The RNA isolation should be done at room temperature and the RNA extracted should be stored at -80 °C. qRT-PCR was utilized to evaluate the subcellular fractions. U6 (nucleus control) and β-actin (cytoplasm control) was set as a control.

### Luciferase reporter assay

Firstly, the potential binding sites of miR-188-5p and LINC00491 were predicted by StarBase3.0 http://starbase.sysu.edu.cn/. Wild-type (wt) LINC00491 and mutant (mt) LINC00491 luciferase vectors were constructed in advanced. At a density of 60%, SW1990 cells were transiently transfected with miR-188-5p negative control or mimics together with LINC00491 wt or LINC00491 mt via Lipofectamine 3000 (Invitrogen) transfection. Then the cells were collected for luciferase activity analysis after 48-incubation.

### RNA immunoprecipitation (RIP) assay

The assay was conducted with the Magna RIP™ RNA-Binding Protein Immunoprecipitation Kit (no. 17-700) purchased from Millipore company. SW1990 cells washed once with PBS were lysed in RIP lysis buffer which contained negative control IgG or Ago2 antibody magnetic beads. The complexes bound by magnetic beads were immobilized with magnet and washed off with wash buffer. After purification of RNA, qRT-PCR was then used to measure the expression of immunoprecipitated LINC00491.

### Pull down assay

Negative control and biotin-labelled miR-188-5p were transfected into SW1990 cells separately via Lipofectamine 3000. About 1×10^7^ SW1990 cells were dissolved in 700 µl lysis buffer with Dynabeads (Invitrogen, USA) after 48h incubation according to the manufacturer's instruments. After being incubated on at 4 °C for 3 h, the beads were washed for 3 times and the retrieved supernatant was prepared for purification of RNAs. LINC00491 was quantified by qRT-PCR.

### Western blotting

Western blotting was conducted as described before 28. In brief, RIPA buffer (APPLYGEN, China) containing 1% protease/phosphatase inhibitor was used for protein extraction. Then 10% sodium dodecyl sulfate polyacrylamide gel electrophoresis (SDS-PAGE) and polyvinylidene difluoride (PVDF) membranes were used to separate and transfer proteins. Then the membranes were subjected to primary antibodies incubation after blocking with 5% milk. Primary antibodies against ZFP91 were purchased from Abcam and β-actin antibodies were purchased from Proteintech. Followed by an hour incubation with secondary antibodies, the bands were observed by the chemiluminescence reagent (Proteintech).

### Mouse xenograft tumor growth assay

12 five-week-old female nude mice achieved from the National Laboratory Animal Center (Beijing, China) were divided into two groups randomly. The assay was conducted according to our previous study 29. The cells of sh-LINC00491 and sh-Con group (about 1×10^7^) were injected into the axillary skin of nude mice from the corresponding groups, respectively. The tumor volume and weight of the two groups were measured every 7 days for four consecutive weeks. Additionally, the expression of LINC00491 and ZFP91 were also detected in the xenografts.

### Statistical analysis

All experiments involving in our research were repeated at least in triple. The data presented as mean ± SD was processed by SPSS software version 26.0 (SPSS Inc., Chicago, IL). The differences between groups were distinguished by Two-tailed Student's T-test or χ^2^ test. Usually, p < 0.05 was considered statistically significant.

## Results

### LINC00491 up-expressed in PC tissues and PC cell lines

**Figure 1:**
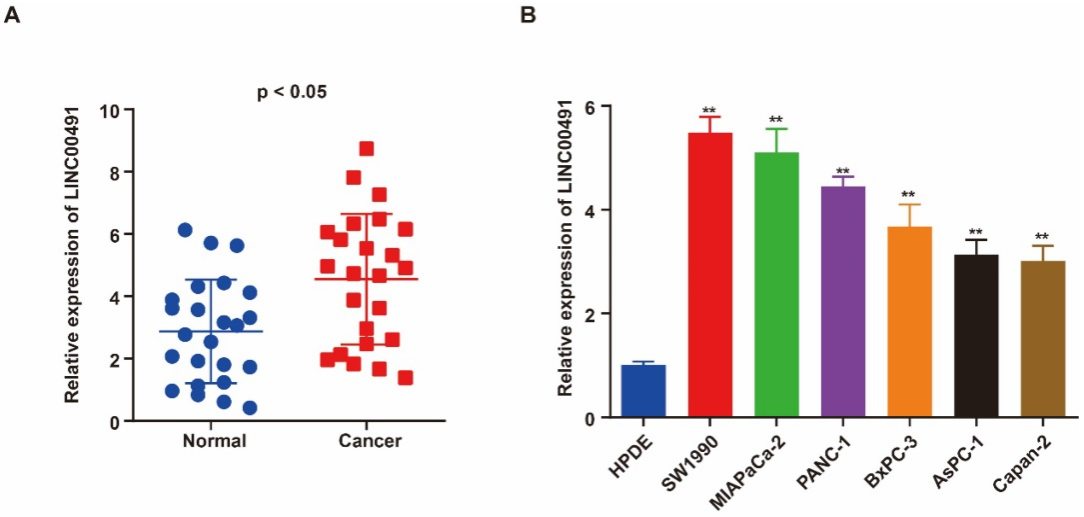
** High expression of LINC00491 in PC cell lines and tissues was associated with poor prognosis. A.** qRT-PCR showed a remarkable increase of LINC00491 in PC tissues. **B.** qRT-PCR detected the expression of LINC00491 in PC cell lines.^ *^p<0.05, ^**^p<0.01, ^***^p<0.001. At least 3 independent experiments were repeated and the results were shown by mean ± SD.

qRT-PCR was performed to assess the level of LINC00491 in PC tissues and adjacent normal tissues. As shown in Figure 1A, LINC00491 in PC tissues expressed highly than the one in adjacent normal tissues. Additionally, the level of LINC00491 in PC cell lines was also evaluated via qRT-PCR. A remarkably increasing LINC00491 was found in PC cell lines compared with HPDE (Figure 1B). Collectively, all the results above suggested that LINC00491 was increased in PC which could be a novel target participated in the progression of PC.

### LINC00491 promoted PC cell migration, invasion and proliferation

**Figure 2:**
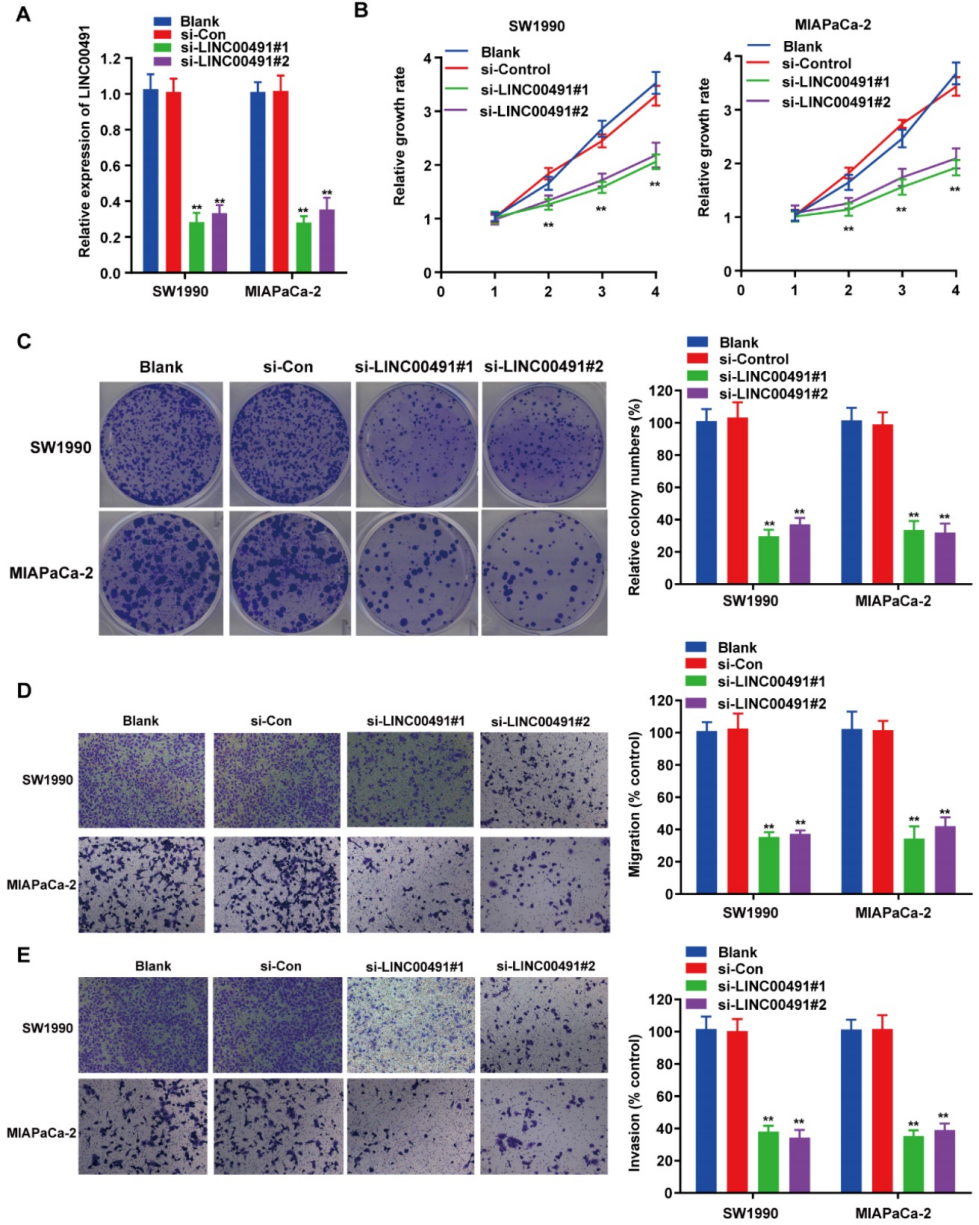
** LINC00491 promoted cell migration, invasion and proliferation of PC *in vitro*. A.** qRT-PCR was performed to validate transfection efficiency of LINC00491 siRNA in SW1990 and MIAPaCa-2. **B.** CCK-8 assay detected the cell proliferation viability of SW1990 and MIAPaCa-2. **C.** Colony formation assays showed the difference of cell proliferation in PC cells with LINC00491 silencing. **D-E.** Transwell assays detected the cell migration and invasion capability of SW1990 and MIAPaCa-2. ^*^p<0.05, ^**^p<0.01, ^***^p<0.001. All experiments were performed independently at least for 3 times and the results were presented by mean ± SD.

To investigate the effect of LINC00491 in PC cell migration, invasion and proliferation, the loss-of-function assays were carried out followed by transfecting LINC00491 siRNA and scramble control into SW1990 and MIAPaCa-2 (Figure 2A). Subsequently, the CCK-8 assay displayed the decrease of LINC00491 in both SW1990 and MIAPaCa-2 inhibited the PC cells proliferation (Figure 2B). Similarly, the colony formation assays also confirmed that the LINC00491 knockdown suppressed the cell proliferation (Figure 2C). Additionally, transwell assays were implemented to illustrate whether silencing LINC00491 could lead to a drop of migration and invasion in PC cell (Figure 2D-E). Concordantly, results implied that the downregulation of LINC00491 were aligned in a decline of migration and invasion ability. Altogether, these findings suggest that LINC00491 knockdown attenuates invasion, migration and proliferation of PC cells.

### The negative interaction between LINC00491 and miR-188-5p

**Figure 3:**
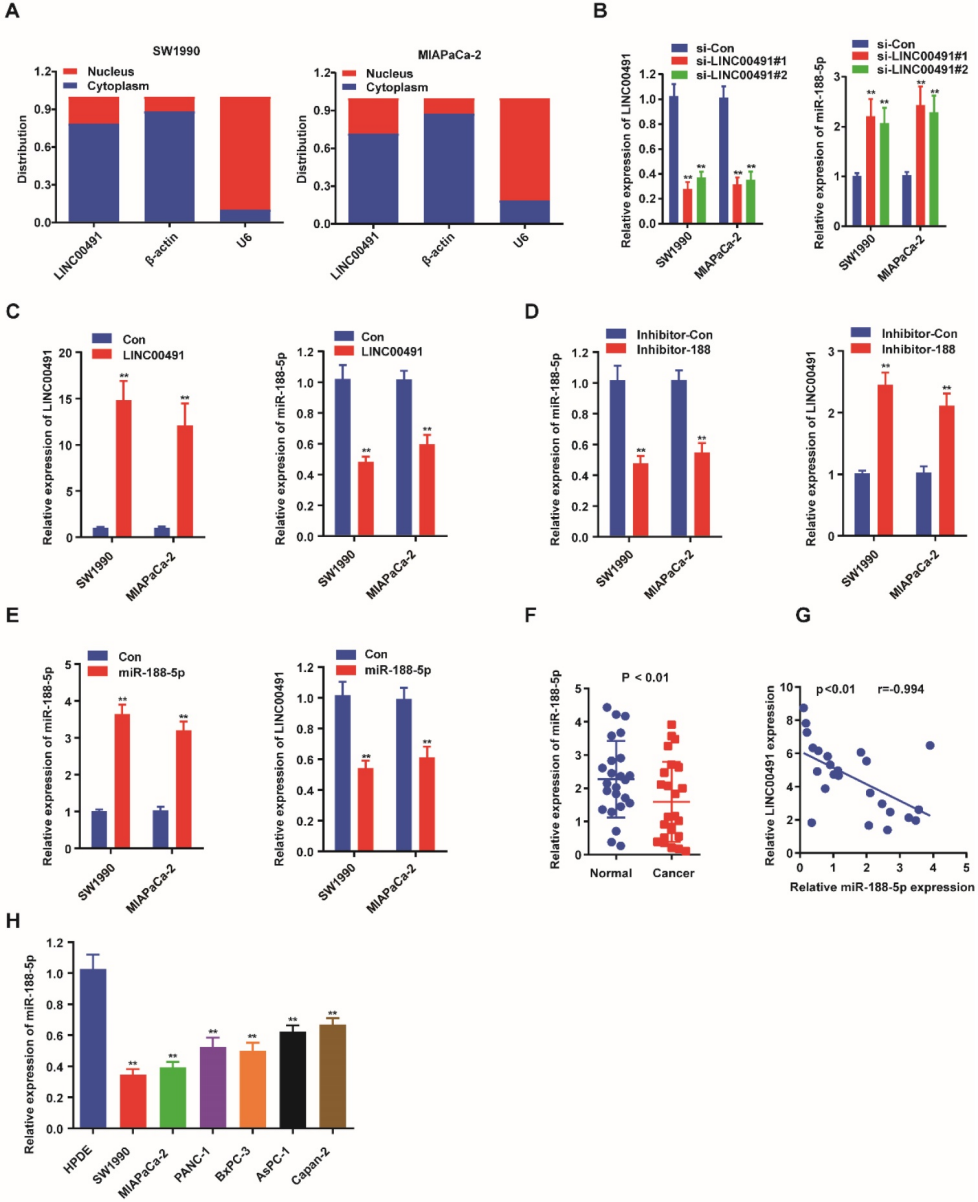
** Reciprocal interaction of LINC00491 and miR-188-5p. A.** The subcellular localization of LINC00491 in SW1990 and MIAPaCa-2 by subcellular fractionation assay. **B-C.** qRT-PCR showed the relative expression of miR-188-5p when down-regulating or up-regulating the expression of LINC00491. **D-E.** qRT-PCR quantified LINC00491 followed by increasing or decreasing the expression of miR-188-5p. **F.** The level of miR-188-5p in PC tissues. **G.** The remarkable negative correlation between LINC00491 with miR-188-5p in PC tissues. **H.** qRT-PCR revealed the expression of miR-188-5P in PC cell lines.^ *^p<0.05, ^**^p<0.01, ^***^p<0.001. At least 3 replicate experiments were performed and final results were presented by mean ± SD.

Previously, many studies showed that lncRNAs could promote the tumor progression via a role as sponge for miRNAs 30. StarBase3.0 http://starbase.sysu.edu.cn/ was used to predict the potential targets between miRNAs and LINC00491, which were examined by qRT-PCR (Table 2). The results revealed miR-188-5p could be a candidate miRNA attributing to its more than twofold expression. Subsequently, we further determined the subcellular localization of LINC00491 to define its potential oncogenic molecular mechanism in PC. The results showed LINC00491 was distributed mainly in the cytoplasm of both two PC cells, indicating LINC00491 may establish its biological role through sponging miRNA (Figure 3A). Subsequently, we detected the level of LINC00491 and miR-188-5p in both SW1990 and MIAPaCa-2 which were transfected with LINC00491 siRNA. Interestingly, the lower expression of LINC00491 was companied with a higher miR-188-5p, which could be reversed by the elevated level of LINC00491 (Figure 3B-C). Furthermore, we further found that while suppressing miR-188-5p with an inhibitor of miR-188-5p could greatly improve the level of LINC00491, boosting miR-188-5p resulted in a dramatically decrease of LINC00491 (Figure 3D-E). All these findings revealed that miR-188-5p might possess an opposite effect in the development of PC compared to LINC00491. In order to confirm this hypothesis, we further used qRT-PCR to explore the expression of miR-188-5p in both PC tissues and PC cell lines (Figure 3F and 3H). Consistently, miR-188-5p shared a similar low expression in both PC cell lines and tissues. Furthermore, the negative correlation between LINC00491 and miR-188-5p in PC tissues was also validated (Figure 3G).

### LINC00491 functioned as a miR-188-5p sponge

**Figure 4:**
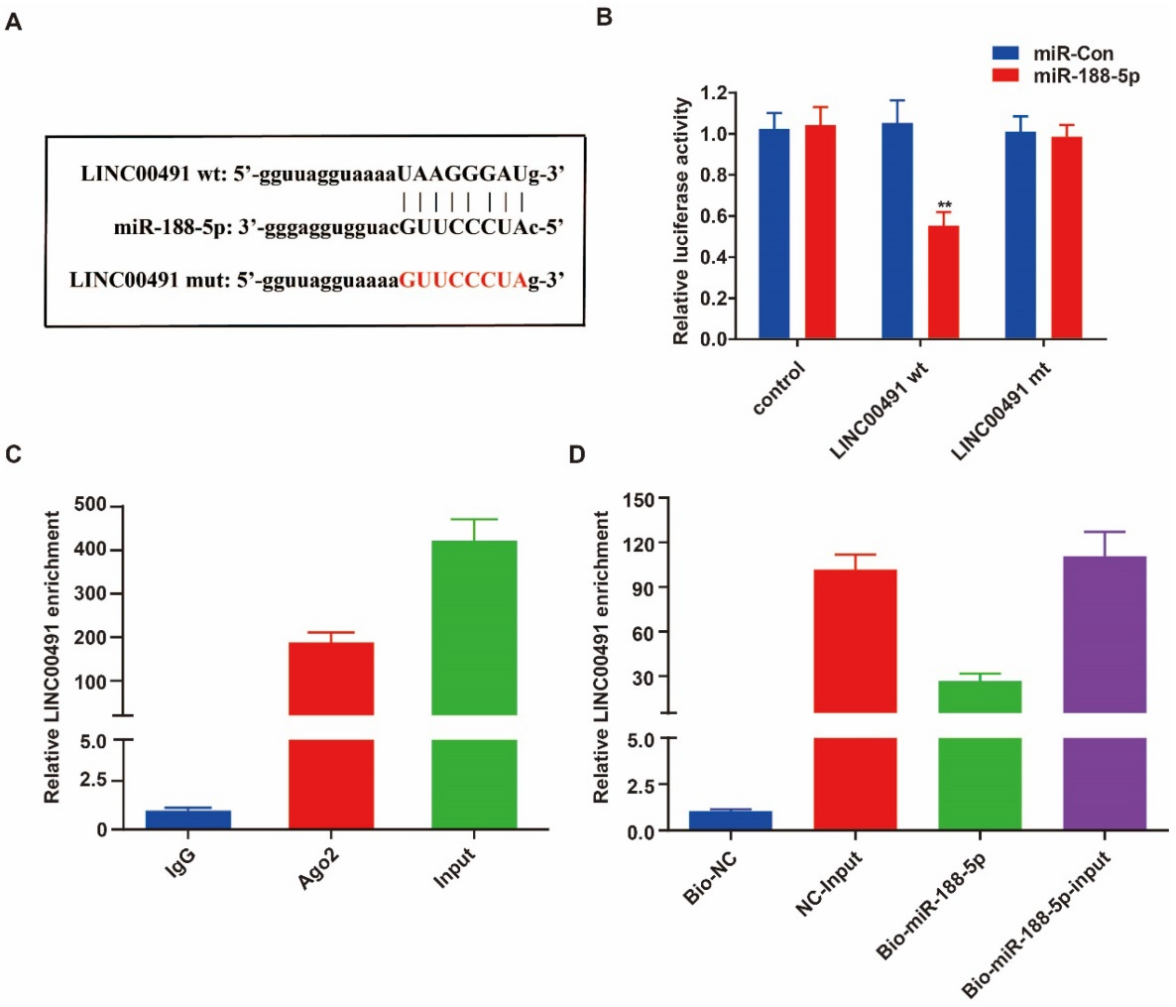
LINC00491 was a sponge for miR-188-5p. A. The sequence of miR-188-5p, wt-LINC00491 and mut-LINC00491. B-C. miR-188-5p was a target of LINC00491 validated by RIP assay and luciferase reporter assay revealed. D. qRT-PCR was conducted to evaluate the relative expression of LINC00491 after pull-down assays. ^*^p<0.05, ^**^p<0.01, ^***^p<0.001. At least 3 replicate experiments were analyzed and the results were presented by mean ± SD.

To determine exact sites between LINC00491 and miR-188-5p, we used the bioinformatics software star Base 3.0 to predict the potential target (Figure 4A). The luciferase assay was then performed and further revealed miR-188-5p can conspicuously decrease the luciferase activity of wild group, however, not of mute group (Figure 4B). In addition, the RIP result demonstrated that LINC00491 was obviously enriched in miRNAs containing Ago2 (Figure 4C). Furthermore, pull down assays further suggested miR-188-5p mimic had pulled down more LINC00491 compared to the negative group (Figure 4D). Overall, all these findings suggest that LINC00491 could act to adhere to miR-188-5p.

### Overexpressing miR-188-5p reversed the anti-tumorigenic effects of LINC00491 knockdown

**Figure 5:**
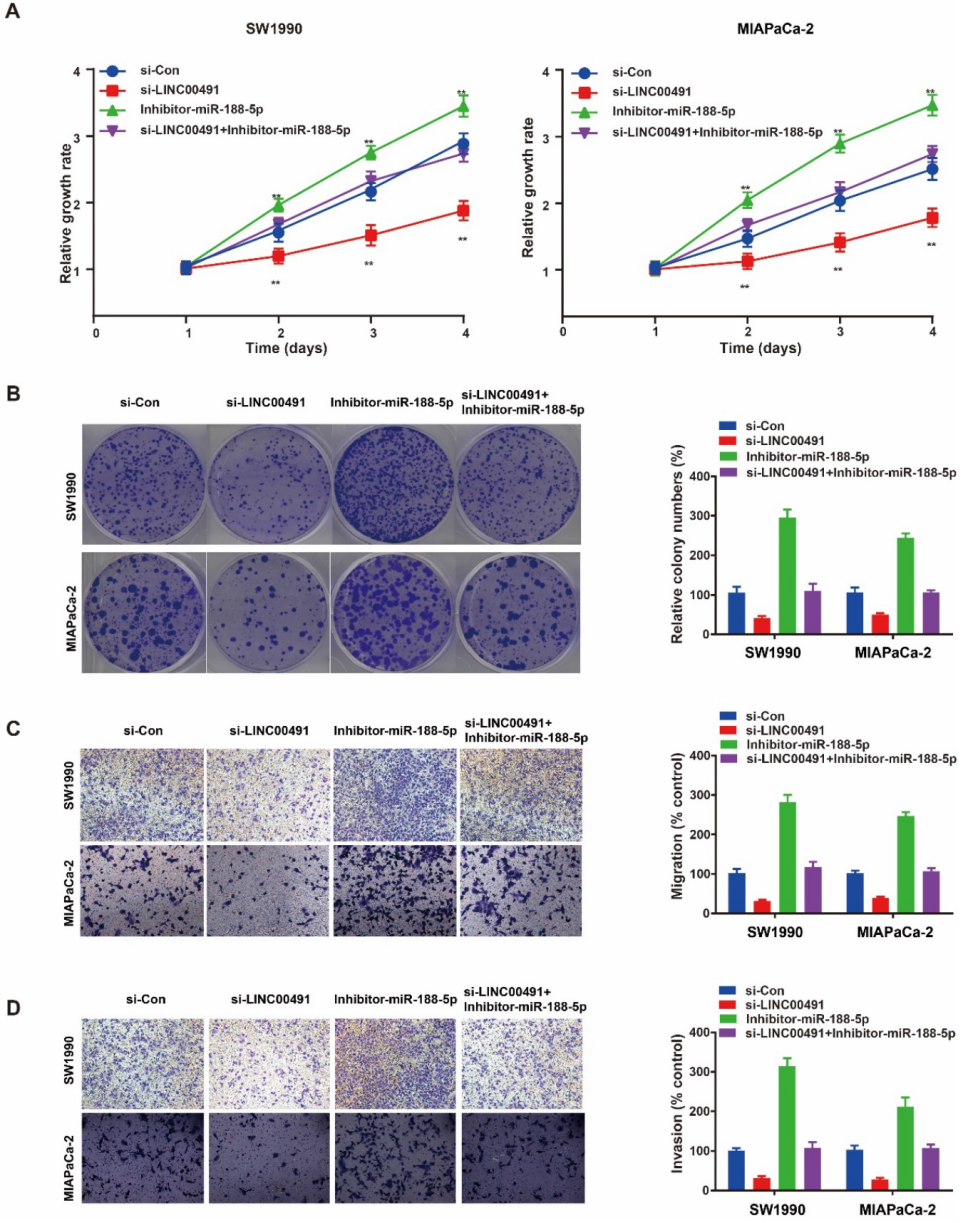
** The reciprocal interaction of LINC00491 and miR-188-5p in PC cells migration, invasion and proliferation. A-B.** CCK-8 assay and colony formation assay were conducted to evaluate the proliferation abilities of SW1990 and MIAPaCa-2 transfected with si-Con, si-LINC00491, miR-188-5p inhibitor, si-LINC00491 and miR-188-5p inhibitor, respectively. **C-D.** Transwell assays indicated the ability of migration and invasion in SW1990 and MIAPaCa-2 under the corresponding treatment. ^*^p<0.05, ^**^p<0.01, ^***^p<0.001. Each experiment was repeated in 3 independent trials and mean ± SD was used to descripted the results.

The recue assay was further performed to identify whether the role of LINC00491 in migration, invasion and proliferation depended on miR-188-5p in PC cells. The CCK-8 assay and colony formation assay showed the decrease of SW1990 and MIAPaCa-2 proliferation ability mediated by knocking down LINC00491 could be reversed by miR-188-5p inhibitor (Figure 5A and B). Additionally, transwell assays showed an inhibition of migration and invasion caused by LINC00491 silencing could also be attenuated by inhibiting miR-188-5p (Figure 5C and D). In summary, LINC00491 partially acts on miR-188-5p to enhance PCs ability of migration, invasion and proliferation.

### Silencing LINC00491 suppressed ZFP91 and enhanced miR-188-5p *in vitro*

**Figure 6:**
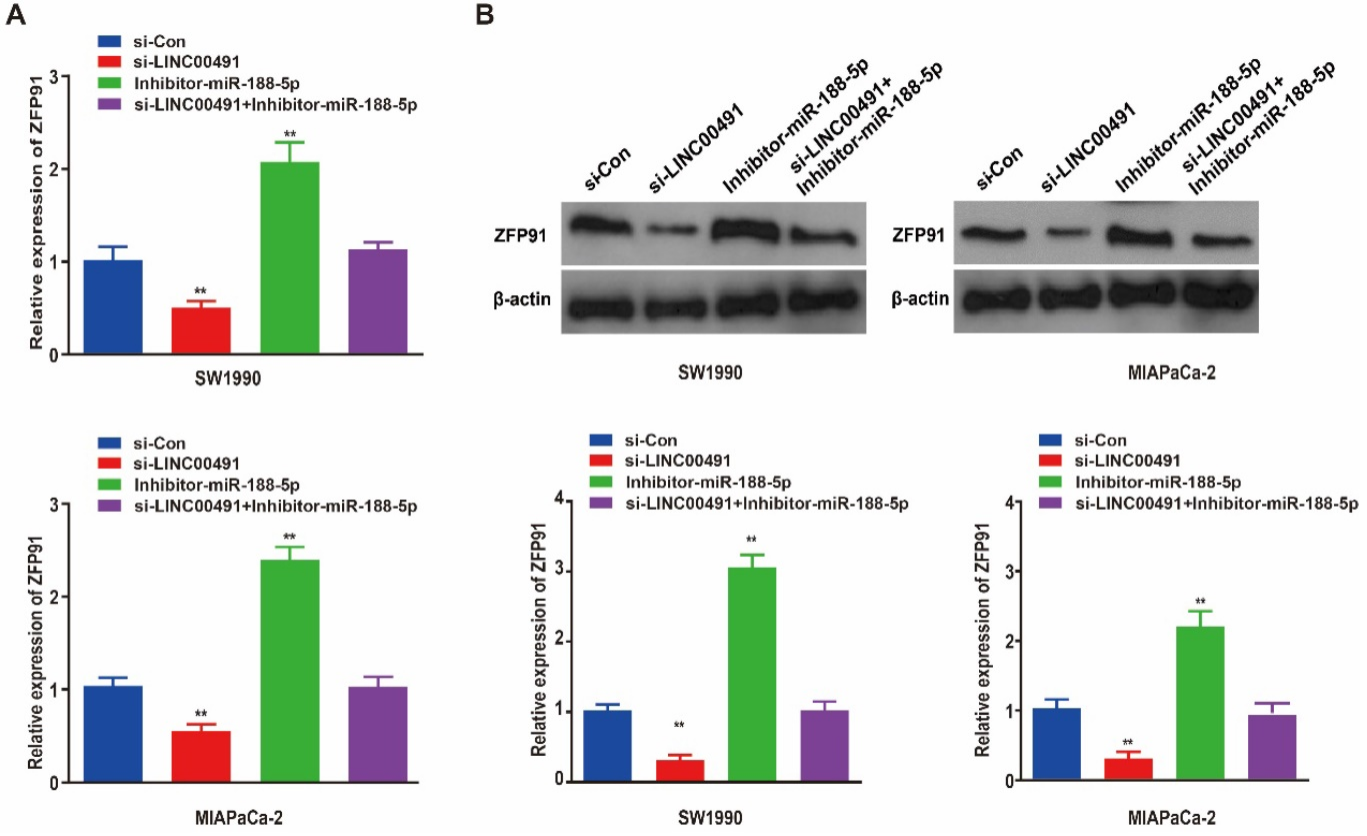
** LINC00491 could up-regulate the expression of ZFP91 through binding and inhibit miR-188-5p. A.** ZFP91 relative mRNA expression levels in SW1990 and MIAPaCa-2 among four different transfections. **B.** ZFP91 relative protein expression levels in the two PC cell lines under different treatment. ^*^p<0.05, ^**^p<0.01, ^***^p<0.001. A minimum of 3 replicates were conducted and the results were shown as mean ± SD.

As previous studies demonstrated, miR-188-5p could regulate tumor development in GC cells by binding with ZFP91 3'-untranslated region (3'-UTR) 31. Therefore, we speculated that miR-188-5p could also regulated PC progression through ZFP91. Subsequently, qRT-PCR and western blotting showed LINC00491 knockdown in both two PC cell lines significantly inhibited ZFP91 expression at the transcription and translation levels followed by an increase in miR-188-5p, which could be abrogated by miR-188-5p inhibitor (Figure 6A and B). Thus far, the above results prove that LINC00491 could inhibit miR-188-5p expression and increase ZFP91 levels.

### LINC00491 promoted tumor growth and regulated ZFP91 protein expression *in vivo*

**Figure 7:**
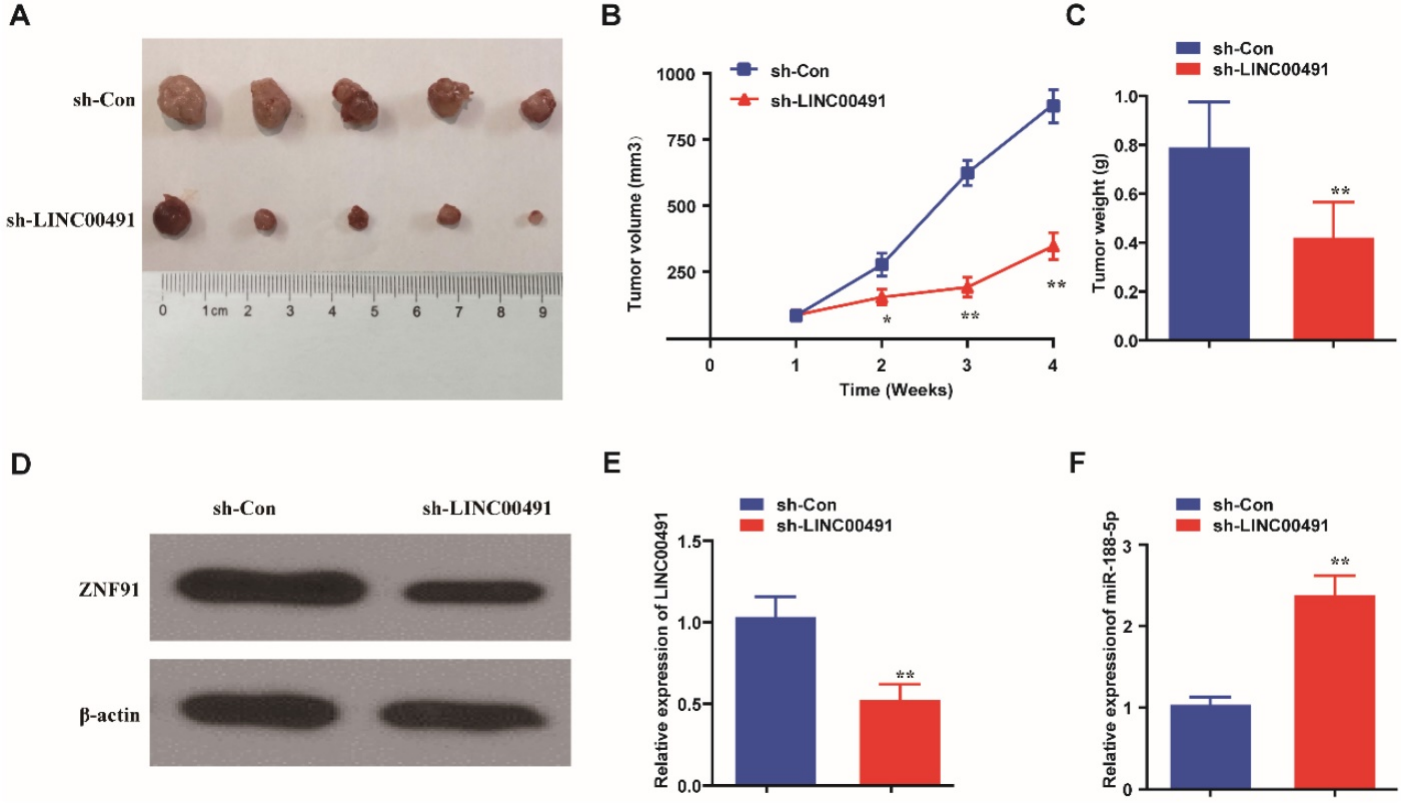
** Down-regulation of LINC00491 suppressed PC tumor growth and reduced the expression of ZFP91. A.** The pictures of the nude mice subcutaneous tumors. **B-C.** The tumor weights and volumes of sh-LINC00491 nude mice group compared to sh-Con mice group. **D.** The relative protein expression of ZFP91 in subcutaneous tumors. **E.** The expression of LINC00491 in subcutaneous tumors. **F.** The expression level of miR-188-5p in subcutaneous tumors. ^*^p<0.05, ^**^p<0.01, ^***^p<0.001. 3 independent trials in each experiment were needed and the data were presented by mean ± SD.

Nude mice xenograft tumor growth assay was further performed to explore LINC00491 effects *in vivo*. SW1990 cells transfected with LINC00491 shRNA or negative control were injected subcutaneously into the armpits of nude mice. After 28 days, the mass tumors were obtained carefully from the mice models (Figure 7A). Results showed that the volume and weight of masses in the LINC00491 shRNA group were less than the one in negative control group (Figure 7B and C). Intriguingly, compared with the control group, the expression of ZFP91 was found be decreased in the tumors in LINC00491 shRNA group, which was consistent with the results in PC cell lines (Figure 7D). Moreover, LINC00491 silencing drastically increased miR-188-5p expression in tumor tissues (Figure 7E and F). Taken together, LINC00491 could modulate the expression of miR-188-5p and ZFP91 to facilitate PC tumor growth.

## Discussion

Recently, PC was considered to be one of the most dangerous cancers that was famous for its poor prognosis despite the use of multi-agent conventional chemotherapy 3. Such unfavorable biological features have fueled ongoing efforts to exploit the tumor therapy, but strategies aimed at deconstructing the pathogenic mechanism and targeting the novel pathways have largely failed 32. It has been reported widely lncRNAs can participate in the tumorigenesis and mediate the initiation and development of many human cancers including human PC 13, 33. Moreover, based on the ceRNAs hypothesis put forward by Tay et al. 19 in 2014, lncRNAs could competitively bind to miRNA to regulate gene expression, a plethora of evidences have thereafter proved the crucial role of miRNAs in alleviating the elevation of lncRNAs during tumor progression, such as PC 34, 35, gastric cancer 36 and ovarian cancer 37, etc. Besides, Lu et al. 38 also illustrated that LINC-DANCR could act as a ceRNA for miR-496 to mediate the expression of mTOR to modulate lung adenocarcinoma progression. Previous researches have shown that the expression of lncRNAs were different in PC which may be associated with the prognosis of PC 39. LINC00491 was one of the new identified lncRNAs that has been firstly determined to predict the survival of breast cancer patients 21. In addition, increasing studies have documented that LINC00491 promoted malignant tumor cellular progression via miRNAs and its downstream axis 20, 26. However, the precise effect of LINC00491 involving in the development of PC is still unknown.

To elucidate this point, the expression level of LINC00491 was evaluated in PC cell lines and tissues. As results showed, the LINC00491 was greatly increased in the cell lines especially in SW1990 and MIAPaCa-2 and PC tissues. Subsequently, loss-of-function studies proved that silencing LINC00491 could inhibit PC cells migration, invasion and proliferation and retard tumor growth. All the results above implied LINC00491 exerted an oncogenic role in the development of PC. To further explored whether LINC00491 can affect the process of PC by interacting with miRNA, we carried out subcellular fractionation assays, which was similar to the research made by Ye et al. 25. Importantly, the results revealed that LINC00491 was mainly located in the cytoplasm, supporting that LINC00491 could be a ceRNA during the course of PC. The mutual negative regulation of LINC00491 and miR-188-5p was thereby further investigated. The results of qRT-PCR showed that overexpressing LINC00491 inhibited miR-188-5p, while silencing LINC00491 upregulated the expression of miR-188-5p, which could be totally reversed when overexpressed or down-expressed miR-188-5p. RIP assay, pull-down assay as well as luciferase reporter assay further confirmed the mutual interaction between LINC00491 and miR-188-5p. Furthermore, recue assay also manifested that miR-188-5p partly inverted LINC00491 function in PC cells proliferation, migration and invasion. Therefore, all of these results validated that miR-188-5p acted as an opponent of LINC00491 to inhibit the PC progression.

It was widely acknowledged that miRNAs repressed protein synthesis by base-pairing to the mRNA 3'-UTR, which usually occurred post-transcriptionally 40. Huang et al. 41 presented an inhibitory effect of decreasing ZFP91 on PC cell invasion and proliferation. Additionally, Yang et al. 42 pointed that miR‑188‑5p could contribute to inhibiting breast cancer the progression by modulating ZFP91 expression. Moreover, the intricate relation of miR-188-5p and ZFP91 was also reported in acute myeloid leukemia 43 and gastric cancer 31. Similarly, the depressed expression of LINC00491 and the increase in miR-188-5p activity were detected, accompanied by decreased ZFP91 expression in tumor of sh-LINC00491 group compared with corresponding control.

To date, the concrete effects of LINC00491 and miR-188-5p are still controversial. In this study, we clarified the possibility of the potential mutual interaction between LINC00491 and miR-188-5p *in vivo* and *in vitro*, respectively. LINC00491 can promote ZFP91 expression through its sponge miR-188-5p ability. Therefore, LINC00491 can be regarded to be a valuable prognostic biomarker for PC therapy in the future.

## Conclusion

In summary, our study provides evidence that LINC00491 can increase ZFP91 expression through sponging miR-188-5p, thereby promoting PC progression. This emphasized the LINC00491/miR-188-5p/ZFP91 axis as one potential treatment strategy for PC.

## Figures and Tables

**Table 1:** Primer information

Gene	Forward primer	Reverse Primer
LINC00491	CTGGCACTCCCTAGTGAGATGAA	GGTTGAGATACACAATGGATTATCCT
miR-188-5p	CCCTCTCTCACATCCCTTGCAT	ATCCTGCAAACCCTGCATGTG
ZFP91	TGAGACCTACAAACCCCACTT	CCTTTTGGGTAAACGTGGACTTT
GAPDH	CGCTCTCTGCTCCTCCTGTTC	ATCCGTTGACTCCGACCTTCAC

**Table 2:** Fold change expression of twenty predicted miRNAs in cells transfected with sh-LINC00491

miRNAs	Fold change	*p* value
miR-384	1.47	˂ 0.01
miR-1294	1.88	˂ 0.01
miR-552-3p	2.13	˂ 0.01
miR-324-5p	2.15	˂ 0.01
miR-188-5p	3.17	˂ 0.01
miR-6866-3p	1.41	˂ 0.01
miR-2681-5p	1.26	˂ 0.01
miR-384	1.17	˂ 0.05

## Abbreviations

PC: Pancreatic cancer
lncRNAs: Long non‑coding RNAs
miRNAs/miRs: MicroRNAs
ceRNA: competing endogenous RNA
LINC00491: Long intergenic non-coding RNA 491
NSCLC: non-small-cell lung cancer
miR-324-5p: microRNA 324-5p
miR-188-5p: microRNA 188-5p
ZFP91: Zinc finger protein 91
DMEM: Dulbecco's Modified Eagle Medium
FBS: Fetal Bovine Serum
sh-RNA: short-hairpin RNA
SDS-PAGE: sodium dodecyl sulfate polyacrylamide gel electrophoresis
PVDF: polyvinylidene difluoride
3'-UTR: 3'-untranslated region
